# Insightful Problem Solving in an Asian Elephant

**DOI:** 10.1371/journal.pone.0023251

**Published:** 2011-08-18

**Authors:** Preston Foerder, Marie Galloway, Tony Barthel, Donald E. Moore, Diana Reiss

**Affiliations:** 1 Biopsychology and Behavioral Neuroscience Program, The Graduate Center, The City University of New York, New York, New York, United States of America; 2 Smithsonian National Zoological Park, Washington, D.C., United States of America; 3 Department of Psychology, Hunter College, The City University of New York, New York, New York, United States of America; Harvard University, United States of America

## Abstract

The “aha” moment or the sudden arrival of the solution to a problem is a common human experience. Spontaneous problem solving without evident trial and error behavior in humans and other animals has been referred to as insight. Surprisingly, elephants, thought to be highly intelligent, have failed to exhibit insightful problem solving in previous cognitive studies. We tested whether three Asian elephants (*Elephas maximus*) would use sticks or other objects to obtain food items placed out-of-reach and overhead. Without prior trial and error behavior, a 7-year-old male Asian elephant showed spontaneous problem solving by moving a large plastic cube, on which he then stood, to acquire the food. In further testing he showed behavioral flexibility, using this technique to reach other items and retrieving the cube from various locations to use as a tool to acquire food. In the cube's absence, he generalized this tool utilization technique to other objects and, when given smaller objects, stacked them in an attempt to reach the food. The elephant's overall behavior was consistent with the definition of insightful problem solving. Previous failures to demonstrate this ability in elephants may have resulted not from a lack of cognitive ability but from the presentation of tasks requiring trunk-held sticks as potential tools, thereby interfering with the trunk's use as a sensory organ to locate the targeted food.

## Introduction

Elephants have large complex brains [1], exhibit complex social behavior [2], show a facility with tools [3], and are generally thought to be highly intelligent [4]. Cognitive studies have demonstrated that elephants are capable of visual symbol discrimination and long term memory [5], means-end recognition [6], relative quantity judgment [7], mirror self-recognition [8], tool use [3], tool manufacture [9], and an understanding of cooperation [10]. Compared to the vast amount of cognitive research in other species, such as primates and birds, a full accounting of the elephant's cognitive abilities is far from complete [11]. However, these studies indicate advanced cognition in elephants. In light of these findings, it is surprising that elephants have been reported to perform poorly in spontaneous or insightful problem solving tasks [12], [13]. This cognitive deficit is unexpected because spontaneous and insightful problem solving has been shown in various species [14]–[17] that show comparable cognitive abilities to elephants. For example, in Köhler's classic studies [17], chimpanzees solved problems suddenly, without trial and error, by using boxes and sticks to acquire bananas hung overhead beyond their reach. Köhler claimed this was indicative of insight.

To further investigate elephants' capacity for insightful problem solving, we initially tested whether three elephants, two adult females, a 33-year-old and a 61-year-old, and a 7-year-old juvenile male (ages at time of testing), at the Smithsonian National Zoological Park, Washington, D.C., USA, would use bamboo sticks as tools to obtain fruit placed out of reach on the opposite side of the bars of their indoor enclosure. At no time did any of the elephants attempt to reach for the food using the sticks, although they manipulated them in tool-like ways within their enclosures: they used the sticks to scratch themselves, hit the floors, walls, and hanging enrichment items, and pried the doors.

**Figure 1:**
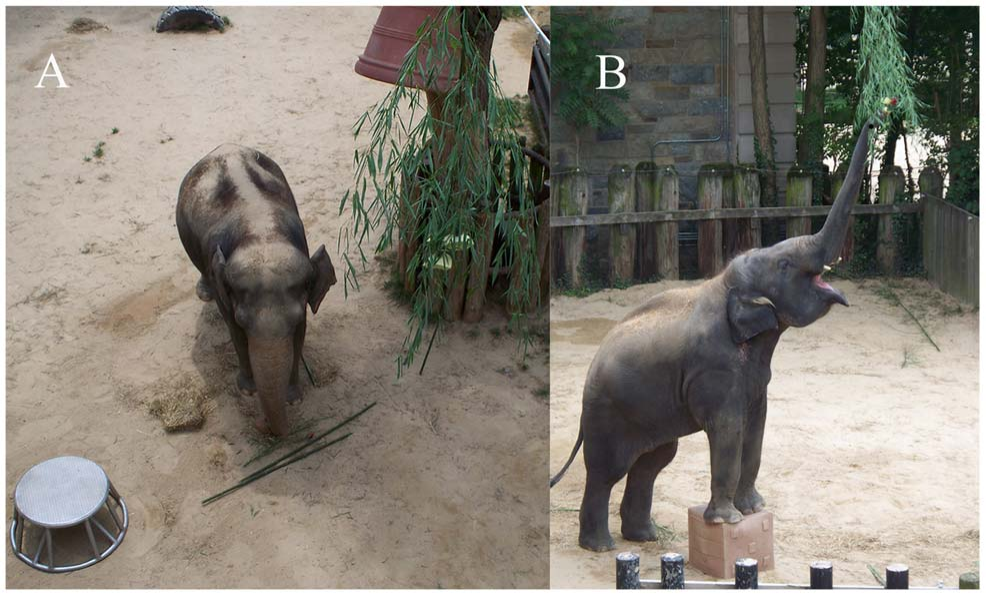
Elephants in Experimental Conditions. (A) An overhead view of the positioning of the elephant tub, sticks and suspended baited branch, with one of the adult female elephants. (B) The juvenile male, Kandula, standing on the cube and reaching for the branch baited with food.

The question arose as to whether the elephants' failures resulted from a lack of problem solving ability, or rather that the bars were impeding the elephants' performance or that the tasks were not ecologically valid. To account for these possibilities, we conducted a second series of tests in the elephants' outdoor yard. See Table S1 for an overview of the experiments. A bamboo branch baited with fruit was hung out of trunk-reach, loosely suspended from an overhead cable. The cable was stretched between the roof of the elephant house and a tree in the elephants' yard. The branches' position varied along the length of the cable for each trial. We provided the elephants with sticks and a large movable object, placed at a distance from the food, which could be moved and used as a tool on which to stand to reach the baited branch. The male elephant, Kandula, was provided with a large, movable plastic cube with which he had previous experience as an enrichment object. The females were provided with an aluminum tub on which they and Kandula had been trained to stand for husbandry examinations. Both objects could accommodate and support the elephants' front two feet. (See Figure 1A for experimental setup.) The females had prior training in pushing large objects (Table S2). Kandula did not receive this training but had previously been observed pushing objects. However, the elephants were neither trained to move objects in order to stand on them nor to use this behavioral sequence to obtain food or other items. Neither the 7-year-old male nor the 61-year-old female had ever been observed moving objects to stand on to obtain food. The 33-year-old female had been observed moving and standing on objects to reach items several times in her adolescence, but not since then, nor since the birth of her son, Kandula.

## Results

### Tool Use

In experiment 1, each elephant was tested individually in sessions lasting approximately 20 min (see Table S1 for exceptions). The elephants were first acclimated to new experimental conditions by giving them easily obtainable (within trunk-reach) baited branches on the cable during three sessions. In experimental sessions, the initial baited branch was obtainable but then placed out of reach in remaining trials.

We ran 28 sessions with the female elephants and 9 sessions with the male. During sessions, the females showed interest in the food by reaching for it but never attempted to use the sticks or to move the tub to obtain it. We discontinued further testing with them.

Kandula also showed interest in obtaining unreachable branches as evidenced by sniffing and trunk-reaching behavior in all sessions (Figure S1) but failed to use sticks as tools to obtain the food. He moved the cube in two of the first six sessions (sessions 1 & 4), but never towards the food. In the first session, Kandula stood on the cube once briefly after rolling it away from the suspended food to an adjacent wall. At no time did he reach for any items while on the cube.

In session 7, Kandula had difficulty removing the first reachable branch from the cable. Four minutes into the session, he obtained the fruit but was unable to pull down the entire branch. He left the food location and returned to the area one minute later, rolled the cube from its original placement to the suspended food's location, stood on it with his front two feet and obtained the branch with his trunk (See Figure 1B and Video S1). Even though he sniffed and reached for the food, he did not use the cube again to acquire food during that session.

**Figure 2:**
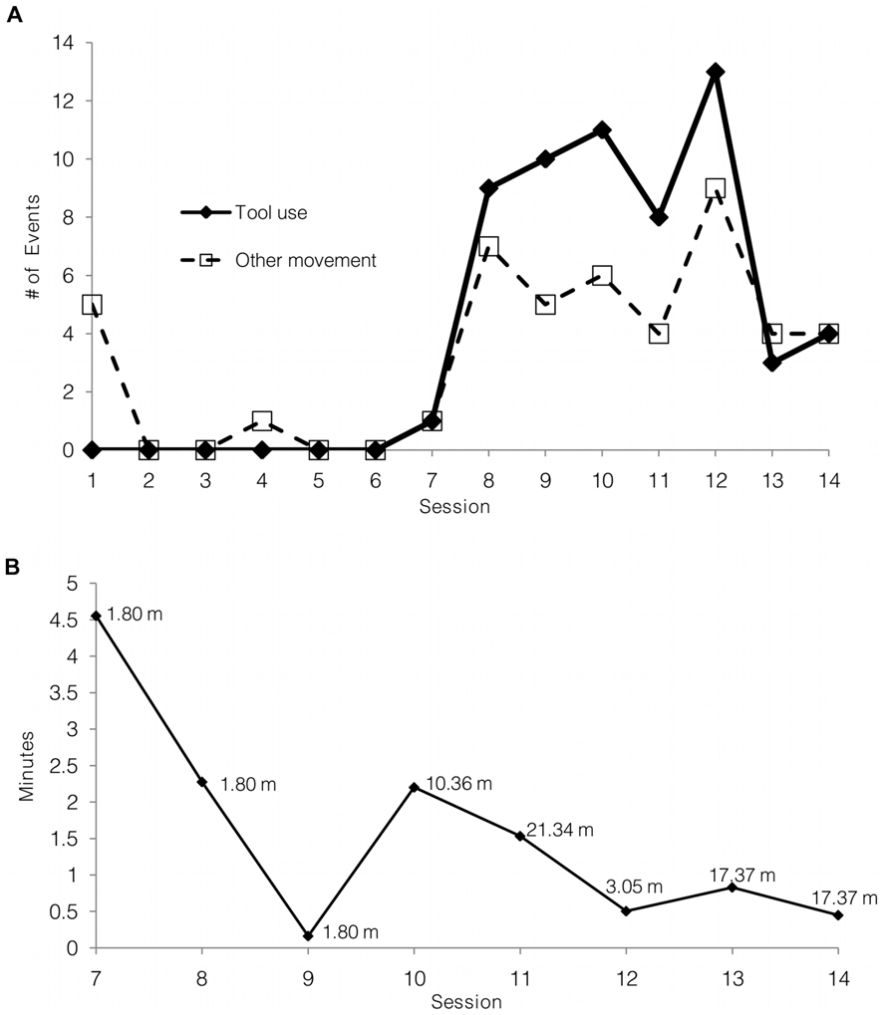
Kandula's Use of the Cube as a Tool. (A) The number of times Kandula rolled the cube in each session that culminated in its use as a tool (i.e., moving the cube, standing on it and reaching for an object) or other movement (e.g., random movement of cube without standing on it) across trials. (B) Latency to the initial rolling of the cube for use as a tool to acquire food in each session. Distances of the initial placement of the cube from food are marked in meters (m). In session 12, the cube was placed on the opposite side of a fence which the elephant could walk around. In sessions 13 and 14, the cube was placed within the entryway to the adjacent yard, a position not visible upon entry from the elephant house.

The next day, in session 8, approximately two minutes after the placement of the first unreachable branch, Kandula rolled the cube to the food area, stood on it, and obtained the food in the same manner as in the previous session. In addition to using the cube for food acquisition, he moved the cube and stood on it to explore the interior of an enrichment object affixed to a tree near the food site. At the session's end, he also rolled the cube to the yard's periphery and stood on it to reach for blossoms on an overhanging tree branch. During this session, he used the cube as a tool to obtain food or other objects a total of 9 times, rolling the cube from 2–10 turns in each effort. Beginning in session 9 all food was hung out of reach in all trials. Kandula used the cube in the same manner to obtain food in subsequent sessions. The location on the cable of the baited branches was changed for each trial in the sessions. Kandula readjusted the position of the cube accordingly. See Figures 2A and B.

### Tool Displacement

We conducted 5 sessions in experiment 2 to test whether Kandula would search for and retrieve the cube when it was placed in different areas of the yard. In the first 3 sessions, the cube was placed at different distances from the food. In each case Kandula exhibited search and retrieval of the cube and then used it to obtain the food. In the last 2 of these 5 sessions, the cube was hidden inside a walled passageway; a position invisible to Kandula upon entry. In the first of these sessions Kandula found the cube and used it to obtain the food. To test if Kandula could recall a previous placement, the next day the cube was again placed in the same hidden location as the previous day. Kandula went directly to the passageway, and used the cube in the same manner (Video S2). Distances and times to initial discovery of the cube are shown in Figure 2B and Figure S2.

### Tool Generalization

In experiment 3, Kandula showed the ability to generalize his tool use to a different object. In a series of four additional sessions we substituted a large tractor tire (previously used as an enrichment item) for the cube. The placement of the tire varied in each session. In three of the four sessions Kandula used the tire as a tool, rolling it to the suspended branches and then standing on it to obtain the food (Video S3). He used it twice in sessions 1 and 4, and once in session 2.

### Stacking

Further inspired by Köhler's chimpanzee studies, in experiment 4 we conducted 8 additional sessions to investigate whether Kandula would stack items to reach food. For these sessions, the baited branches were hung at a height that could be reached by stacking three butcher block cutting boards or by the use of other objects. In addition, the elephant was given sticks and other enrichment items. Kandula first touched several items and then moved two items, a plastic disk and a block under the suspended branches, placing one front foot on each in an unsuccessful attempt to reach for the branch. He solved the problem in an unexpected novel manner, moving and standing on the object closest in size to the absent cube, a large ball. Standing on unstable platforms such as this had not been previously observed. He repeated this behavior 9 times during this session. During the session's last minutes, Kandula picked up a block ∼2 m from the food and placed it directly on top of a block that he placed under the food in a previous attempt. He stood on the stacked blocks and attempted to reach the food but was unsuccessful (Video S4). He stacked two blocks again in the second and sixth sessions but each time his trunk was several inches from the food.

## Discussion

These results provide experimental evidence that an elephant is capable of insightful problem solving through tool use. Evidence for this ability is indicated by the suddenness of Kandula's problem solving behavior without evidence of prior trial and error learning. His persistent use of this problem solving technique in subsequent sessions and his transference to other objects is consistent with the definition and other criteria that some have set for insightful problem solving [13], [18]. Elephants in the field [19] and those in this study have been observed standing on stationary objects to attain items. However, Kandula's movement of the cube for use as a platform to attain otherwise unreachable food was a novel and spontaneous solution to the problem. It could be argued that the elephant had prior training in a component of the novel problem solving task, standing on an object. However, the sequence of behavior exhibited by Kandula, moving the cube and standing on it to reach food, constitutes a more complex series of events that cannot be accounted for by past training. Kandula's use of the cube and other objects is also consistent with a current definition of tool use [20] in that the object was moved and effectively oriented by the user prior to use to alter the position of the user itself. The onset of the elephant's stacking behavior may not be indicative of insight as it was preceded and followed by trials in which he persisted in trying to use single blocks. Kandula's behavior suggests, however, that he was actively trying to use different objects and strategies for food acquisition. Each time a method was unsuccessful, he switched strategies. Table 1 presents the sequential order of behavior that Kandula exhibited.

**Table 1:** Sequential order of Kandula's behavior in Experiment 4, Session 1.

Time in Session	Behavior	Successful
21 s	Rolls ball to food. Places foot on ball but does not stand on it.	No
2 min 42 s	Brings block next to disc (previously placed by elephant), stands with one foot on each and reaches for food.	No
2 min 52 s	Rolls ball to food, stands on ball and reaches for food.	Yes
3 min 9 s	Rolls ball to food, stands on ball and reaches for food.	Yes
3 min 38 s	Rolls ball to food, stands on ball and reaches for food.	Yes
4 min 8 s	Rolls ball to food, stands on ball and reaches for food.	Yes
5 min 22 s	Rolls ball to food, stands on ball and reaches for food.	No
7 min 28 s	Carries single block to food, stands on block and reaches for food.	No
13 min 20 s	Rolls ball to food, stands on ball and reaches for food.	No
13 min 34 s	Rolls ball to tree, stands on ball and reaches towards tree.	No
14 min 20 s	Carries single block to food, stands on block and reaches for food.	No
16 min 45 s	Gets off block and reaches for food with feet on ground.	No
23 min 43 s	Carries single block, stacks on the stack (previously placed by elephant), stands on it and reaches for food.	No
23 min 46 s	Kneels on back legs, placing head and trunk in a more vertical, steps off stack, reaches for food, places foot on stack and reaches for food.	No
24 min 24 s	Stands on stack and reaches for food.	No
26 min 20 s	Rolls ball to food, stands on ball and reaches for food.	Yes
27 min 11 s	Rolls ball to food, stands on ball and reaches for food. Attempt aborted due to end of session.	N/A

List of all behaviors exhibited by Kandula to reach for food or other objects and his success in food acquisition in the first block stacking session. Time in Session refers to the elapsed time from the beginning of the session. At 5 m 22 s, Kandula had removed enough leaves from the branch so that it was no longer reachable. Branch was replaced at ∼10 min.

We believe that the problem in previous studies has been in treating the elephant trunk as a grasping appendage analogous to a primate hand. Although the trunk is a highly manipulable appendage, in food foraging its function as a sensory organ may take precedence. The elephant has an extraordinary sense of smell [21], and the tip of the trunk is as highly enervated as a human fingertip [22]. The trunk has been described as a “refined eating tool. [21]” It is a superb appendage to locate, examine and acquire food and other objects as it provides the animal with the interaction of olfactory and tactile information. The elephant's eye has a fovea directed at the end of the trunk [23] further facilitating the sensory interaction with visual information. When a stick is held in the trunk, the tip is curled backwards and may be closed, prohibiting olfactory and tactile feedback. These deficits might not deter the elephant from using a trunk-held tool for other tasks but they may inhibit the use of such tools to acquire food. Although elephants have shown the greatest frequency and diversity of tool use of any non-primate mammal, they use tools primarily for skin care [3]. In an extensive review of elephant tool use, only one example of an elephant using a trunk-held tool to acquire food was found [3], [24]. Kandula's placement of the cube to use as a platform brought his trunk closer to the food allowing him to take advantage of his trunk's sensory abilities. We posit that previous failures to observe insightful problem solving in elephants [12] is not indicative of a lack of cognitive ability but rather is due to the reliance on problem solving tasks that precluded the use of the trunk as a sense organ.

The neuromorphology of the elephant brain has been implicated by some researchers [12] as the cause for the apparent lack of insightful problem solving ability. The elephant brain has been described as having neurons that are larger and less densely packed than the primate brain [25]. Hart *et al* [12] have speculated that elephant cortical neurons may have longer axons traversing more distant cortical regions. It has been further hypothesized that this may result in decreased local compartmentalization resulting in increased time of information processing. Such slower processing has been posited as an explanation for the elephant's poor performance in cognitive tasks. However, a more recent study [26] has shown that the elephant's neural morphology is more complex than previously thought, possibly suggesting greater integration of information, enabling advanced cognitive abilities such as insightful problem solving.

Whether the behavior described herein is truly “insightful” is, of course, a point for discussion. Insight has been controversial since the word *Einsicht* was first translated from Köhler's German [17].Thorpe later defined insight as the “sudden production of new adaptive responses not arrived at by trial behavior…or the solution of a problem by the sudden adaptive reorganization of experience [18].” Although this definition has been used as a standard, some have found it lacking in not addressing the possible cognitive processes underlying insight [27] such as the use of mental trial and error in problem solving [28]. Others have proposed that the term insight implies a causal explanation and should be abandoned entirely in behavioral studies [29], [30]. Gallistel *et al*. [31] have proposed a model of learning based on evidence gathering in which an animal changes abruptly to a different strategy when evidence exceeds a decision threshold. Durstewitz *et al*. [32] have supported this model in rats by demonstrating neurological evidence corresponding to the sudden shifts in behavioral strategies as seen in insightful problem solving. They concluded that their results “support the idea that rule learning is an evidence based decision process perhaps accompanied by moments of sudden insight.”

Although the specific cognitive processes underlying the precipitousness of Kandula's behavior remain in question, this study demonstrates that elephants are capable of insightful problem solving. When given the proper circumstances, elephants, like humans and several other species, can demonstrate “aha” moments.

## Materials and Methods

### Ethics Statement

All experiments were reviewed and approved by the Institutional Animal Care and Use Committee of Hunter College, City University of New York (Approval #DR-insight 6/11-01), decision reviewed and accepted by the Smithsonian National Zoological Park.

### Subjects

We tested three Asian elephants (*Elephas maximus*) currently housed at the Smithsonian National Zoological Park (NZP), Washington, D.C. The group was composed of: Ambika, female, approximate date of birth – 1/1/1948, weight – approx. 3240 kg. She arrived at NZP in 1961. Ambika was captured in the Coorg Forest, India at about 8 years old. She was trained and used as a work elephant for about 2 years before coming to the U.S.A. Shanthi, female, approximate date of birth – 1/1/1976, weight – approx. 4050 kg. She arrived at NZP 12/30/76. Originally from Sri Lanka, Shanthi was found in a well at approximately 3 months old and raised in a Sri Lankan elephant orphanage. Kandula, male, born 11/25/01 at NZP of Shanthi through artificial insemination, weight – approx. 2250 kg. He has lived with his mother since birth.

All three elephants were tested in Experiment 1. Only Kandula was tested in Experiments 2–4. Animals were maintained under normal care and feeding protocols. None were food deprived for this study. Elephants had been trained in a number of behaviors. See Table S2.

### Facilities

Elephants were housed in the NZP Elephant House. Facilities consisted of 6 stalls 7.9 m across with walls on three sides and vertical bars facing the public area. Experimental sessions were conducted in the elephant yard exterior to the elephant house. This yard consists of two adjoining yards separated by a fence that is open on either end, measuring 24.4 m×25.9 m. This yard also adjoins a larger yard separated by a large motor operated gate that was closed during sessions. There are two trees within the yard, as well as trees bordering the yard. There is a large bathing pool as well as enrichment items in the yard. The public had a view of this yard at the opposite side from where experimental sessions were conducted. See Figure S2 for photo of yard.

### Experiment 1

#### Apparatus

A 7.62 m cable was run from the roof of the elephant house to a tree in the yard. The height of the cable was 6.25 m above the ground at its center. A movable shuttle attached to a rope pulley was positioned on the cable. Lengths of leafy bamboo with fruit attached at the bottom were hung from the shuttle by a trimmed branch so that they could be pulled or knocked off. Fruit was attached to the bamboo by impaling it on branches with leaves. Fruit is a preferred food and each length of bamboo had three pieces of fruit that was varied among melons (cantaloupe or honeydew), apples, bananas, and oranges.

In the yard were placed four 1.80 m lengths of bamboo sticks, two leaning against the tree and two on the ground beneath the food. In addition, Ambika and Shanthi were given an elephant “tub,” a round aluminum stand 0.61 m tall and 0.75 m in diameter. Kandula was given a 0.61 m plastic cube that supported his weight. Neither of these items were novel. Ambika and Shanthi had been previously trained to stand on the tub (see Table S2.). Kandula had previous experience with the cube as an enrichment toy. Different items were used because 1) the cube might not support the weight of the larger elephants and 2) Kandula, being more playful, might pick up the metal tub and throw it, creating dangerous containment issues. The platforms were placed approximately 1.80–3.66 m from the food placement. Exact distance and position varied.

#### Procedure

Sessions were 20 minutes in duration. Later sessions, with the two females together, were 30 minutes in duration. If food and sticks were still available, sessions were sometimes extended because of other responsibilities of the keeping staff. Overall, sessions with individual elephants averaged 26 minutes. Trials were determined by completion of the task. Experimenters were positioned on the roof for both observation of the experiment and placement of the bamboo. In pre-testing, three sessions were used to acclimate each elephant to feeding from the overhead bamboo stalk and determine the proper height to put the food out of reach. No sticks or other objects were in the yard during these sessions.

Each experimental session began with the elephant receiving one length of bamboo with fruit that could be reached with the trunk. The bamboo was pulled into position approximately halfway along the cable using the pulley. The position of the food was varied. After the elephant took the first bamboo branch, it was replaced by a length of bamboo that was out of trunk-reach unless the subject stood on a platform. If this branch was taken, or needed replacement, it was replaced with another similar piece until the end of the session. After 16 sessions, it was decided that if the two females were tested in the yard together, the session could be extended from 20 to 30 minutes. Twelve subsequent sessions were conducted in this manner.

### Experiment 2

#### Apparatus

Same as in Experiment 1.

#### Procedure

Both sticks and the cube were available. At the beginning of each session the cube was placed in a different position. See Figure S2. All positions are described from the experimenter's vantage point on the roof.

Session 1: The cube was placed 10.4 m to from the food. Session 2: The cube was placed approximately 21.3 m away from food by a pool. Session 3: The cube was placed 3.1 m away from the food on the opposite side of a fence. Session 4: The cube was placed 17.4 m away from the food within the entryway to the adjacent yard, a position not visible upon entry from the elephant house. Session 5: The cube was placed in same position as in session 4.

### Experiment 3

#### Apparatus

Food presentation same as in Experiment 1. The cube was replaced by a large tractor tire, 1.27 m in diameter, 0.53 m thick. Four sticks were provided as in the previous experiments.

#### Procedure

Same as in previous experiment. Four sessions were conducted. In each session, the tire was placed in a different position in the yard.

### Experiment 4

#### Apparatus

Food presentation same as in Experiment 1. The cube was replaced by three wooden butcher block cutting boards, 0.61 m×0.46 m×5.7 cm. In addition to the boards, four other items previously used for enrichment were provided. In the first session, these items were a blue plastic disc, 5.0 cm×0.60 m, a green plastic cone, 0.61 m, a hollow plastic ball, 0.46 m, and a hard plastic ball, 0.76 m. Four sticks were provided as in the previous experiments. After the first session, the hard plastic ball and the hollow plastic ball were replaced with a 0.61 m round flat blue barrel lid.

#### Procedure

There was some doubt if Kandula could handle or move the flatter butcher blocks. Therefore one session was conducted with a single block in the yard with Kandula, without food or other objects. He showed facility in moving and carrying the block. Subsequent sessions were run without altering the blocks.

Nine sessions were conducted. In the first session, the blocks were interspersed with the enrichment items, positioned in a semicircular array. In subsequent sessions, the items were lined up approximately 4.6 m from the food. The order of blocks and enrichment items was randomized. The baited branches were hung at a height that would necessitate Kandula stacking three blocks to reach them.

## Supporting Information

Figure S1 **Kandula's Interest in Food.** Number of times the elephant either sniffed at or reached for food without acquisition in each session. The elephant acquired the food in sessions 7, 8, and 9.
(TIF)

Figure S2 **Elephant Yard with Positions and Distances of Cube Placement.** The yard is 25.91 m wide×24.38 m deep. The arrow marked with a star indicates the food placement. Sessions are indicated by the numbers in arrows. The elephant entered from elephant house door at center bottom of photo.
(JPG)

Table S1 **Overview of Experiments.**
(DOC)

Table S2 **List of commands and trained behaviors for each elephant.**
(DOC)

Video S1 **This video shows the elephant's first use of the cube as a tool to acquire food.** (QuickTime; 2.0 MB).
(MOV)

Video S2 **This video shows the elephant retrieving the cube from a non-visible placement during the second session in which the cube was placed in this position.** The video starts from elephant's entrance at the beginning of the session (QuickTime; 4.9 MB).
(MOV)

Video S3 **This video shows the elephant employing a tire as a tool to obtain food.** (QuickTime; 2.0 MB).
(MOV)

Video S4 **This video shows the elephant stacking two blocks in an attempt to acquire food.** (QuickTime; 4.6 MB).
(MOV)
