# Supplementary figures and images for: Insightful Problem Solving in an Asian Elephant
Source: PLoS One. 2011 Aug 18;6(8):e23251. doi: 10.1371/journal.pone.0023251 (PMC3158079; doi:10.1371/journal.pone.0023251)

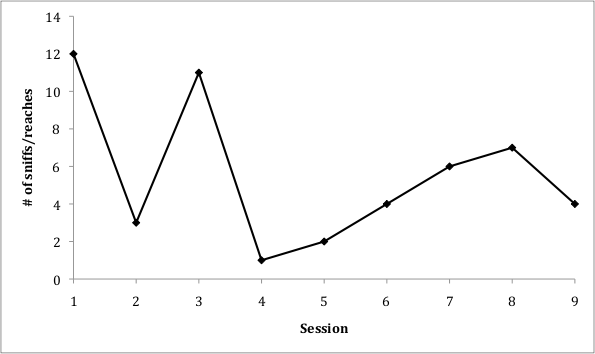

Supplement: Figure S1 — Kandula's Interest in Food. Number of times the elephant either sniffed at or reached for food without acquisition in each session. The elephant acquired the food in sessions 7, 8, and 9. (TIF) [file pone.0023251.s001.tif]

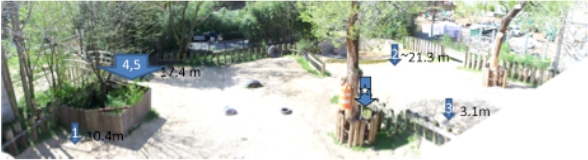

Supplement: Figure S2 — Elephant Yard with Positions and Distances of Cube Placement. The yard is 25.91 m wide×24.38 m deep. The arrow marked with a star indicates the food placement. Sessions are indicated by the numbers in arrows. The elephant entered from elephant house door at center bottom of photo. (JPG) [file pone.0023251.s002.jpg]
