# Supplementary material for: Insightful Problem Solving in an Asian Elephant
Source: PLoS One. 2011 Aug 18;6(8):e23251. doi: 10.1371/journal.pone.0023251 (PMC3158079; doi:10.1371/journal.pone.0023251)
Supplement: Table S1 — Overview of Experiments. (DOC) [file pone.0023251.s003.doc]

**Table S1. Overview of Experiments**

| Experiment | Protocol | Elephant | # of Sessions/ Period of Days |
| --- | --- | --- | --- |
|  |  |  |  |
| 1 | Tool use with cube | K | 9/12 |
| 1 | Tool use with tub | S | 16/ 19 |
| 1 | Tool use with tub | A | 16/19 |
| 1 | Tool use with tub | S & A | 12/17 |
| 2 | Cube displacement | K | 5/14 |
| 3 | Tire as tool | K | 4/5 |
| 4 | Block Stacking | K | 9/9 |
