# Supplementary material for: Insightful Problem Solving in an Asian Elephant
Source: PLoS One. 2011 Aug 18;6(8):e23251. doi: 10.1371/journal.pone.0023251 (PMC3158079; doi:10.1371/journal.pone.0023251)
Supplement: Table S2 — List of commands and trained behaviors for each elephant. (DOC) [file pone.0023251.s004.doc]

**Table S2*.* List of commands and trained behaviors for each elephant.**

| Command | | Description | Elephant |
| --- | --- | --- | --- |
| All right | | release from previous command | All |
| At ease | | trunk at rest, straight down | All |
| Back | | move back in a straight line | All |
| Blow | | exhale forcefully through trunk | All |
| Bow | | bend left wrist back and touch it to the ground with trunk up | All |
| Bring | | bring object being held to handler | All |
| Corners | | raise opposite front and rear legs | Ambika, Shanthi |
| Crawl | | move forward in either the stretch or kneel positions | Kandula |
| Cross | | cross left legs in front of right legs | All |
| Down | | lateral recumbency | All |
| Ears | | present ears forward | All |
| Foot-on front leg | | wrist to elbow parallel to ground | All |
| Foot-on rear leg | | foot to knee parallel to ground | All |
| Give | | hand object to handler, hold it until taken | All |
| Half | | half way between stretch and down | All |
| Harness | | lower head for harness | Ambika, Shanthi |
| Command | | Description | Elephant |
|  | |  |  |
| Head | | lower head while front feet are extended out | All |
| Here | | move designated body part towards handler | All |
| Hold | | do not release grip (with mouth or trunk) | All |
| Kneel | | down on front wrists | Kandula |
| Leave | | drop whatever is in trunk, or don’t touch | All |
| Lift | | front foot raised as high as it can go, pad up, slightly outward, rear legs tucked | All |
| Line | | elephant stands facing handler and/or in specific order (hierarchical) | All |
| Move | | move forward in a straight line | All |
| No | | stop unwanted behavior | All |
| Open | | open mouth for tooth inspection | All |
| Over | | move away | All |
| Pad | | front foot bent back at wrist, pad showing | All |
| Pick | | lift an object with trunk | All |
| Place | | stay in a designated area | All |
| Command | | Description | Elephant |
| Put it away | | put tire on top of bars | Shanthi |
| Relax | | relax trunk muscles for handler manipulation | All |
| Salute | | on rear knees with left front leg raised with trunk up | All |
| Same | | raise both legs on same side of body | All |
| Shake | | shake head | Ambika, Kandula |
| Side | | touch mid section of body to target | All |
| Stretch | | sternal recumbency | All |
| Tail | | grab tail of another elephant, | All |
| Also:Tail | | touch base of tail to target | All |
| Touch | | touch trunk to handler hand or target | All |
| Trunk | | curl trunk up to touch forehead | All |
| Tub | | climb onto object - stump or balance beam | All |
| Turn | | move from a center axis pivot | All |
| Wiggle | flap ears | | Shanthi |
| Wait | do not proceed (Only for calves or elephants not yet trained to steady, a transition to place or steady) | | All |
